# Modulation of cytotoxic and genotoxic effects of nanoparticles in cancer cells by external magnetic field

**DOI:** 10.1186/s12645-014-0002-x

**Published:** 2014-06-26

**Authors:** Jyoti Shaw, Sufi O Raja, Anjan Kr Dasgupta

**Affiliations:** Department of Biochemistry and Centre for excellence in Biomedical Engineering and Systems Biology, University of Calcutta, Kolkata, 700019 India

**Keywords:** Static magnetic field, Magnetic nanoparticle, DNA damage, Membrane potential, Cell viability, Normal cell and cancer cell

## Abstract

Magnetic nanoparticles are well known for anticancer activity by deregulating cellular functions. In the present study, cellular effects of low strength static magnetic field (SMF) were explored. How nanoparticles affect the cellular response in presence and absence of static magnetic field was also studied. Peripheral blood mononuclear cells (PBMC) and human lymphoma monocytic cell line U937 were chosen as representative normal and cancer cells models. The two effects we would like to report in this paper are, DNA damage induced by SMF of the order of 70 mT, and alteration in membrane potential. The other notable aspect was the changes were diametrically opposite in normal and cancer cell types. DNA damage was observed only in cancer cells whereas membrane depolarization was observed in normal cells. Iron oxide nanoparticles (IONP) and gold nanoparticles (AuNP) were also used for cellular response studies in presence and absence of SMF. The effects of the magnetic nanoparticle IONP and also of AuNP were sensitive to presence of SMF. Unlike cancer cells, normal cells showed a transient membrane depolarization sensitive to static magnetic field. This depolarization effect exclusive for normal cells was suggested to have correlations with their higher repair capacity and lesser propensity for DNA damage. The work shows cancer cells and normal cells respond to nanoparticle and static magnetic field in different ways. The static magnetic induced DNA damage observed exclusively in cancer cells may have therapeutic implications. From the conclusions of the present investigation we may infer that static magnetic field enhances the therapeutic potentials of nanoparticles. Such low strength magnetic field seems to be a promising external manoeuvring agent in designing theranostics.

**Electronic supplementary material:**

The online version of this article (doi:10.1186/s12645-014-0002-x) contains supplementary material, which is available to authorized users.

## Background

Cancer cells and normal cells differ in their cell-cell communication [1], characteristic cell death [2] repair mechanisms or other cellular activities [3]. They exhibit considerable genomic instability and immortality. Designing an agent that serves as a discriminator for cancer cells and performs targeted killing of the same, has been a primary objective in theranostics [4]. This was a term coined to express simultaneous execution of therapy and diagnostics. Most of the theranostic studies are motivated by exploitation of tumor-targeted nanoparticles and manipulation of their cellular interaction [5]. Use of magnetic nanoparticle [6] as theranostic agent has recently become popular as their presence or absence can be realized by magnetic imaging and characterised by relaxometry or superconducting quantum interference device (SQUID) measurements [7]. Secondly, they can be used as hyper-thermic agent in presence of fluctuating external magnetic field [8]. It may be noted that cell specificity of nanoparticles [9], is an important factor determining the success of a given theranostic agent. An ideal nano-therapeutic or theranostic agents would be one which may show specific toxicity towards cancer cells but is relatively inert towards normal cells. When amenable to external manoeuvring the therapeutic potential of the nano agent can be further enhanced. Commonly, basis for such external manipulation is hyperthermia, in which local heating causes cell death. The hyperthermia, in turn can be induced in cells by combination of nanoparticle and radiofrequency field [10] or laser radiations [11]. Very little insight however exists on how an external static magnetic field [12] would affect cancer cells. While static magnetic fields (SMF) are unlikely to cause heating, it may be important to study the cytotoxic effects of static magnetic field on DNA damage and other intracellular processes.

In this context it may be also noted that nanoparticles alone can damage cells by targeting DNA. In a recent report [13] indicated that nanoparticles can damage DNA of cancer cells without actual penetration in the nuclear milieu and they perform such acts by invoking new cellular signalling pathways. In combination with SMF this damage may be modulated, and this is one of the perspectives explored in this paper.

It may be recalled at this stage that original interest for application of nanotechnology in cancer biology, stemmed from the expectation that cancer cells would show higher uptake of nano-materials, as there is constant demand for nutrient supply to pace up with their high metabolic rate, a point that may have some supporting evidences [14]. At the same time we cannot neglect the effect of nanomaterials on normal and healthy cells present in the local milieu. The literatures are present about the toxicity of nanomaterials on cancer cell line as well as primary cells. This is the second perspective dealt in this paper. What we need to understand with better depth is how the responses of normal cells and cancers cells towards individual theranostic components, differ.

In this paper we had shown that there could be a number of routes like DNA damage, membrane potential change and cellular damage by which nanoparticles can induce cancer cell specific toxicity. As model systems normal and cancer cells we considered peripheral blood mononuclear cells (PBMCs) [15], and a suspended histiocytic lymphoma cell line, U937 [16]. Both are suspended cells and may have identical local environment as doubling time for U937 is approximately 96 hours and PBMCs do not multiply in absence of any mitogen (like phytohaemagglutinin). Thus these two cell types are similar for comparison in the present experimental condition. We have further considered standard nano particles, like iron and gold and a supposedly neutral agent, low strength static magnetic field (70 mT) as theranostic discriminators. This study, other than opening up some comparative systemic differences in normal and cancer cells, may help in unfolding biophysical processes specific to such differential responses.

The question why SMF is expected to affect cellular function can be answered in two ways. Classically, cellular components are diamagnetic in nature [17] and the question is how cells and cells exposed to nanoparticles respond to such diamagnetic perturbations. The second way of interpreting the SMF effect reported in various cellular contexts, though in a rather incoherent manner, is that SMF imparts a weak spin perturbations and such weak perturbations are amplified by the coherence existing in various subcellular components [18].

While the paper does not resolve the exact mechanism, but it leaves ground for exploring some definite questions regarding the static magnetic field guided modulations of cellular poalrization and DNA damage, one being linked to the energetics of the cells and the other linked to cell death.

## Methods

### Synthesis of nanoparticles

Gold nanoparticle (AuNP) was synthesized according to Turkevich’s method [19-21]. 0.256 mM Chloroauric acid was reduced by 1.2 mM sodium citrate in the boiling condition with continuous stirring. Brick red color colloidal suspension of AuNP was formed. Similarly Iron oxide nanoparticle (IONP) was synthesized by double reduction of ferrous and ferric salts [22]. The nanoparticles were then characterised by dynamic light scattering (DTS Nano zeta-sizer), scanning electron microscopy (SEM) and UV–vis Spectroscopy (see Additional file 1: Figure S1).

### Ethics statement

Blood was collected from healthy human subjects with informed consent and the matter was cleared by ethics committee of Nilratan Sarkar Medical College, Kolkata.

### PBMCs isolation and culture

Peripheral blood mononuclear cells (PBMCs) were isolated from peripheral blood of healthy human volunteers by venipuncture in a sodium citrate vial. The protocol by Ivan J. Fuss [23] was followed with few modifications. Diluted blood (1:1 diluted with PBS) was carefully overlaid on the layer of ficoll-hypaque (Histopaque-1077 from SIGMA Aldrich) and centrifuged at 1600 rpm for 30 min at room temperature. After centrifugation, the buffy coat layer of PBMCs was carefully aspirated to a fresh tube, which was then washed with PBS and centrifuged at 1200 rpm for 10 min. This step was repeated twice to remove the trace of platelets and obtained an almost pure population of PBMCs. The viability count was performed using trypan blue exclusion test in a haemocytometer. This method yields approximately more than 95% viable and PBMCs. PBMCs were then suspended at 1 × 10^6^ cells/ml in RPMI 1640 complete medium (GIBCO BRL) supplemented with 10% FBS, 1% Pen-strep and 2 mM L-glutamine. PBMCs were then incubated in a 5% CO_2_ incubator at 37°C.

### Treatment of PBMCs (normal cells) and U937 (cancer cells) with different nanoparticles (GNP and IONP) both in presence and absence of an external static magnetic field (70 mT)

PBMCs were treated with GNP and IONP separately in a 12 well tissue culture plate. Three different doses (100 μg/ml, 10 μg/ml and 1 μg/ml) of both nanoparticle (NP) were given to cells in RPMI complete medium and incubated in 5% CO_2_ incubator at 37°C. In a duplicate plate and separate incubator, Static magnetic field (SMF) exposure was given to cells so that unexposed cells were not affected by such external perturbation.

Similarly U937 cell lines was cultured and plated in a 12 well tissue culture in RPMI 1640 complete medium at a concentration of 1×10^6^ cells/ml at 37°C in 5% CO_2_ incubator. Three different doses of GNP and IONP were also given to cells. SMF exposure was given at a separate incubator in a duplicate plate. The work had been repeated more than thrice and results shown were mean of all the experiments.

### DNA damage assay by single cell Gel electrophoresis (SCGE)

SCGE or Comet assay was performed on both normal and cancer cells after treatment with respective controls (cells not exposed to nanoparticles and SMF). Detection of DNA damage in the PBMCs was done by SCGE or Comet assay using protocol developed by Singh and Tice et al. method [24]. To brief, PBMCs were mixed with low melting agarose (LMA) and layered over pre-coated glass slides. Then a third layer of LMA was applied on it. Then slides were dipped in lysis buffer and incubated overnight at 4ºC. Afterwards slides were kept in alkaline electrophoresis buffer for 20 min and then electric field 24 V or 300 mA is applied. After that slides were neutralised using neutralising buffer and stained with EtBr. The slides were then manually scanned under fluorescence microscope. The images of comets were scored by the comet program COMET SCORE.

### Flow cytometry analysis of cells treated with Nanoparticles

After treatment with nanoparticles and exposure to SMF, normal and cancer cells were assayed by flow cytometry in BD Verse instrument. Mitochondrial membrane potential and cellular viability of the cells were analyzed using JC-1 [25] and Propidium Iodide (PI) probe [26] respectively.

#### Alteration in mitochondrial membrane potential (ΔΨ) using JC-1 probe

JC-1 (5,5′,6,6′-tetrachloro-1,1′,3,3′-tetraethylbenzimidazolcarbocyanine iodide) is a lipophilic fluorescent probe which is used to evaluate the status of ΔΨ in a cellular system. It is a cationic lipophilic membrane permeable dye that emits fluorescence in both FITC and PE channel. JC-1 is a monomeric molecule that forms aggregates (J-aggregates) at high dye concentration. J-aggregates emit fluorescence in both FITC and PE channel but JC-1 monomers give fluorescence only in FITC. In a live cell JC-1 penetrates the plasma membrane and accumulates within mitochondria as J-aggregates. ΔΨ of such cells are polarised and give fluorescence at both FITC and PE channels. In a depolarised cell, JC-1 monomers start to leak from mitochondria to the cytosol and thus give only FITC fluorescence. Cells with high PE and high FITC intensity are polarised and cells with only high FITC fluorescence are depolarised.

#### Cellular viability assessment using propidium iodide dye

Propidium Iodide (PI) is a DNA staining dye and it is widely used to determine cell viability by flow cytometry. After treatment with two type of nanoparticles separately, PI was given to about 50,000 cells at concentration of 5 μg/ml and incubated for 5 min at room temperature and analyzed immediately by BD Verse. PI has absorption maximum at 535 nm and gives maximum fluorescence at 617 nm and is captured at PE channel. The data was analyzed by Flowjo, software version 10.0.6.

#### Gating nomenclatures

Flow cytometry data were analyzed in flowjo software. In each sample 10,000 cells were acquired which contain desired population of more than 50%. The desired population was the lymphocyte enriched viable population in case of PBMC cells. This lymphocyte enriched population was referred to as L1. Similarly in case of cancer cells (U937) 10,000 cells were acquired which mainly contains viable population. In order to exclude cellular debris, viable population was gated as P1. This LI and P1 population was further sub-divided into L2 and L3, and P2 and P3 in the analysis of JC-1 data. L2 and P2 are population of polarised cells and L3 and P3 were population of depolarised cells in normal and cancer cells respectively.

#### Data analysis

FCS files were transformed into csv format by flowjo program. Then the csv files were analyzed in a MATLAB platform. The 2D data (e.g. FSC, SSC) was converted to a 2D grid in which the matrix elements indicate the events occurrence density. To compare the effects of field (or any other particles) the control and the experimental matrices were dumped into two independent colour planes. While pure colours represented either control or experimental (red and green in case of SMF). The superposed regions represented in the grid points having overlap data (points where experimental data is similar to control). The result is represented as RGB image. The image is rotated in such a way that the XY-axes are the conventional XY co-ordinates and not the image axes. The scale of image is represented along a normalized logscale.

#### Determination of ΔΨ from JC-1 data using matlab program

ΔΨ of the cells are determined using matlab computer program with the help of the following MATLAB function (MATLAB 9.0, MATHWORKS USA):

Function psi = jyoti_potcal(x,y)

% x -- > array of monomer counts (FITC Fluorescence)

% y -- > array of dimer counts (PE Fluorescence)

% the function returns the single cell membrane potential

% the dye dimer is sensitive to membrane potential

% x + x →y

% dimensionalize to express fractional concentration

x = x/sum(x);

y = y/sum(y);

keq = y./(x.^2);

R = 8.314462; F = 9.6485e + 004; T = 300;

% assume the dye is in Nernst equilibrium

% Electrochemical potential = 0

% The membrane potential is equivalent to chemical potential difference of the dye

% This is the free energy (delta G = −RT ln keq) scaled by Faraday’s constant

psi = (R*T/F) * log(keq);

% express psi in millivolts

psi = psi*1000;

## Results

### Particle characterization

AuNP synthesized was found to have hydrodynamic diameter in the range of 30-40 nm. This was further confirmed by scanning electron microscopy or SEM. Tthe UV–vis absorption spectra of the same give maxima at 520 nm. This peak maxima is characteristically given by the 20-30 nm size gold nanoparticles. The actual particle might be further smaller in size. The stability of the particle was given by zeta potential. The mean zeta potential was -41 mV, which indicate that nano-colloidal suspension was well stable. Similarly, IONP was having hydrodynamic diameter of 60-70 nm and zeta potential of -48 mV. The figures and graphs are given in the Additional file 1.

### Normal and cancer cells - SMF effect

**Figure 1:**
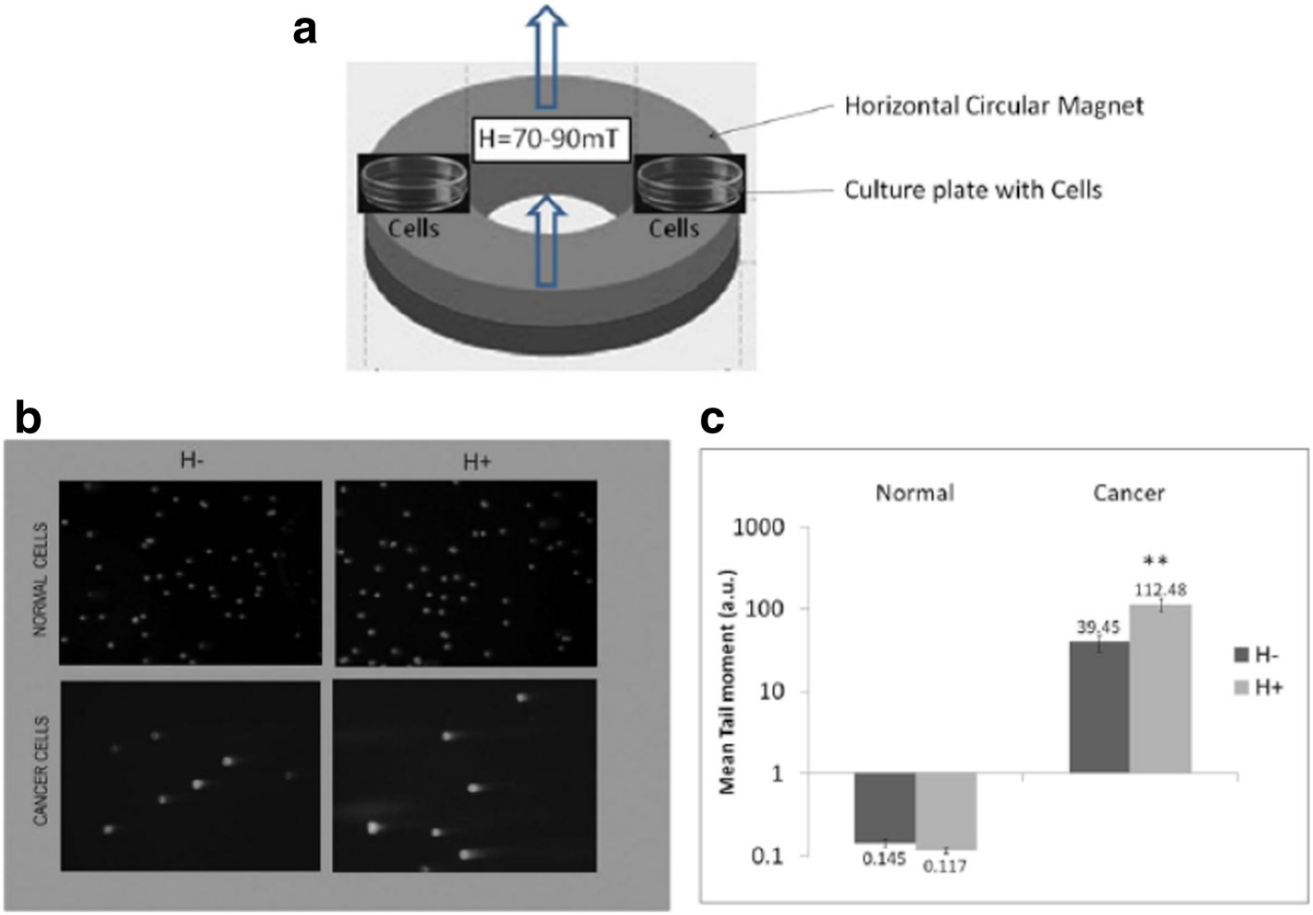
**Comparison of DNA damage in normal and cancer cells in response to SMF.** Panel **a**, shows schematic diagram of the exposure set-up. Both normal and cancer cells were incubated in absence and presence of SMF (70 mT) in ambient growth condition for 12 hours in separate culture plates prior to the comet assay. Panel **b**, Images of comet assay done on respective normal and cancer cells after exposure to SMF. In case of cancer cells number of comets formed is more in presence of SMF (+H). Panel **c**, Compares DNA damage in cells in terms of mean tail moment. T-statistic gives significant P-value. All values are Mean ± S.E., **denotes P ≤ 0.001.

Two types of cells one cancer cells (U937) and other normal cells (PBMCs) were assayed to study the effect of SMF on DNA of these cells. The upper panel of Figure 1, illustrates the setup using which the magnetic field perturbation was given. The lower left panel compares the SMF effect on normal and cancer cells using Comet assay. It may be seen that mere presence of magnetic field results in loss of DNA integrity in cancer cells but no such effects were visible in normal cells. The bar diagram in the lower right panel of Figure 1, compares mean tail moments of comets formed in the two cell types in presence and absence of SMF. Tail moment gives the estimation of the percentage of DNA present in the tail of a comet of a cell as observed under fluorescence microscope obtained after comet assay. The log scale illustrates the dramatic difference in the SMF response of the two cell types. The normal cells show almost no SMF induced DNA damage (as measured by mean tail moment for control: 0.145, for SMF treated cells: 0.117), while the cancer cell show DNA damage; for control: 39.45 and for SMF treated cells: 112.48. Statistical tests (t-stat) indicate that the difference is significant for cancer cells (H = 1, p = 0.002, t = 3.9009) and insignificant for normal cells (H = 0, p = 0.5, t = 0.65), H representing true or false nature of the non-null hypothesis.

**Figure 2:**
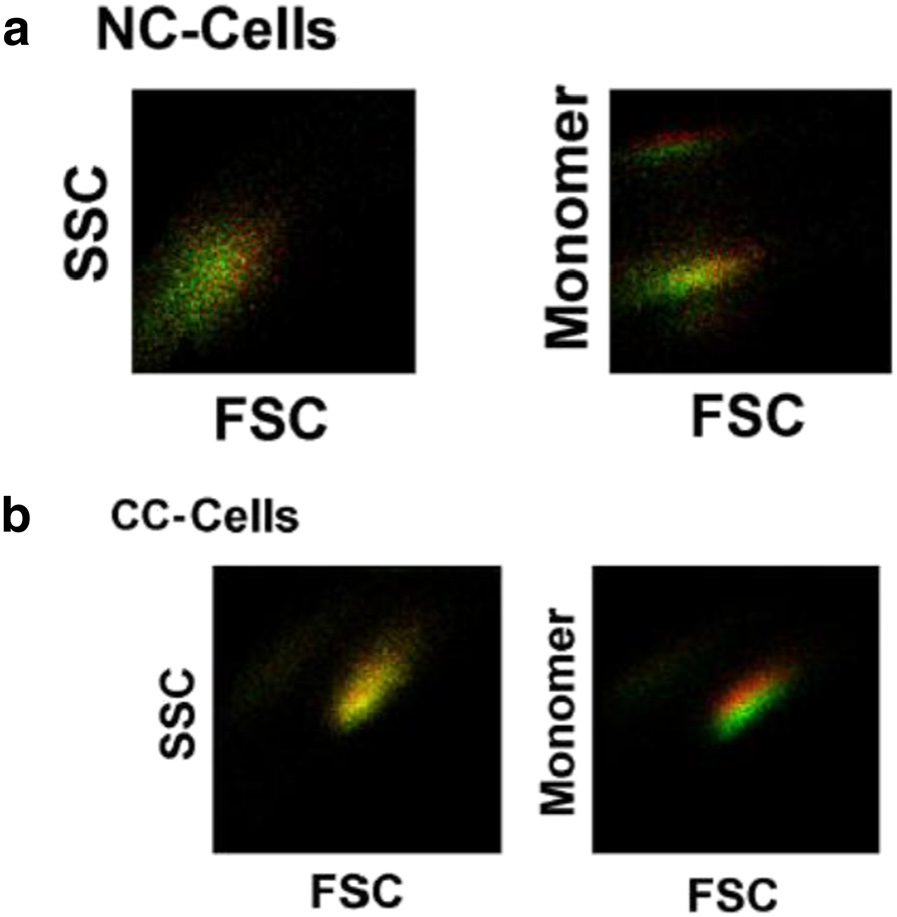
**Shift in ΔΨ in normal cells and cancer cells in response to SMF.** The pseudo images are density plot in which log scale values are rescaled within 0 to +1. The details of the image analysis is given in Methods section. Absence and presence of SMF are represented by red and green color. Panel **a** shows normal cells (NC-Cells) and panel **b** shows cancer cells (CC-Cells). SMF did not affect morphology in both cell type but unlike normal cells, it did affect ΔΨ in cancer cells.

In Figure 2, flow cytometry analysis of normal cells (top panel) and cancer cells (bottom panel) are shown after analysis in Matlab program. The images illustrates that morphology of both cell types are not altered by the presence of SMF but mitochondrial membrane potential is slightly affected by the presence of magnetic field in cancer cells but normal cells remain unchanged. This gives evidence of greater penetration of SMF in cancer cells as compared to normal cells.

### DNA damage by Nanoparticles and by combination of SMF and nanoparticles

**Figure 3:**
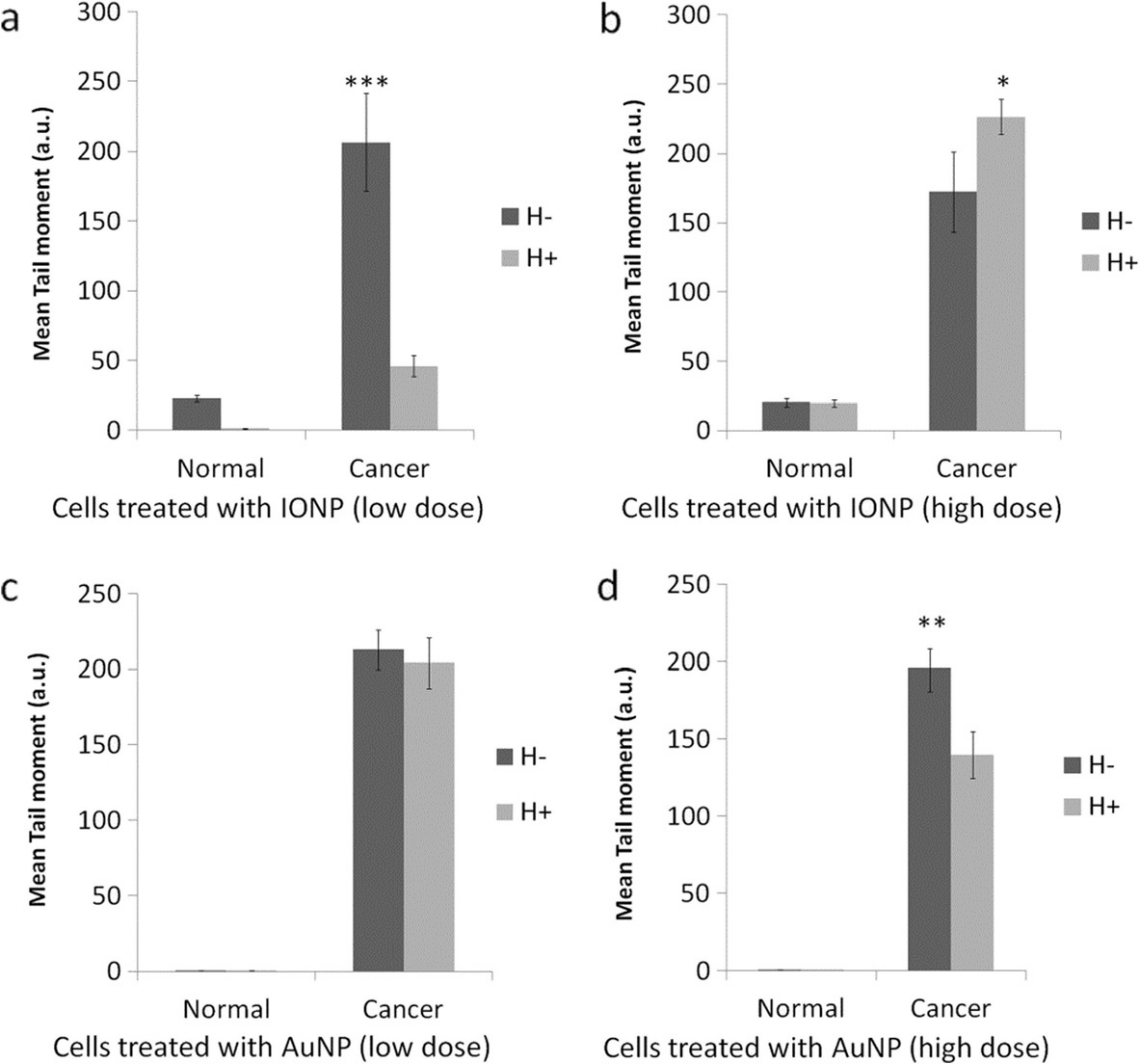
**Comparison of DNA damage in normal and cancer cells by the combined effect of of nanoparticle and SMF.** Panels **a** and **b** shows IONP treated cells and Panels **c** and **d** shows AuNP treated cells. Both IONP and AuNP caused significant DNA damage in cancer cells. The extent of DNA damage in normal cells is very less in normal cells as compared to cancer cells. AuNP did not cause any damage in normal cells (mean tail moment <1). T-statistic compares mean tail moment of cells between unexposed and exposed to smf. *P ≤ 0.05, ***P ≤ 0.0001. All values are Mean ± S.E.

In Figure 3, the effect of AuNP and IONP on DNA damage is presented in the form of mean tail moment. IONP caused higher damage to DNA of cancer cells compared to that of normal cells. However, the combined effect of IONP and SMF is also examined in this section. SMF increased DNA damage in cancer cells at higher dose of IONP but had reverse effect at lower dose. This reversibility was also seen in case of normal cells. Synergistic effect of SMF was found in AuNP treated cancer cells. Cancer cells treated with both AuNP and SMF showed higher damage to DNA compared with normal cells. The presence of field increased the genotoxic potential of AuNP at high dose whereas at low dose of same nanoparticle had no additional effect of SMF on DNA damage of cancer cells as seen by comet assay. In case of normal cells, AuNP did not cause any DNA damage to these cells as mean tail moment is < <1.

### Cell viability in response to Nanoparticles/SMF

**Figure 4:**
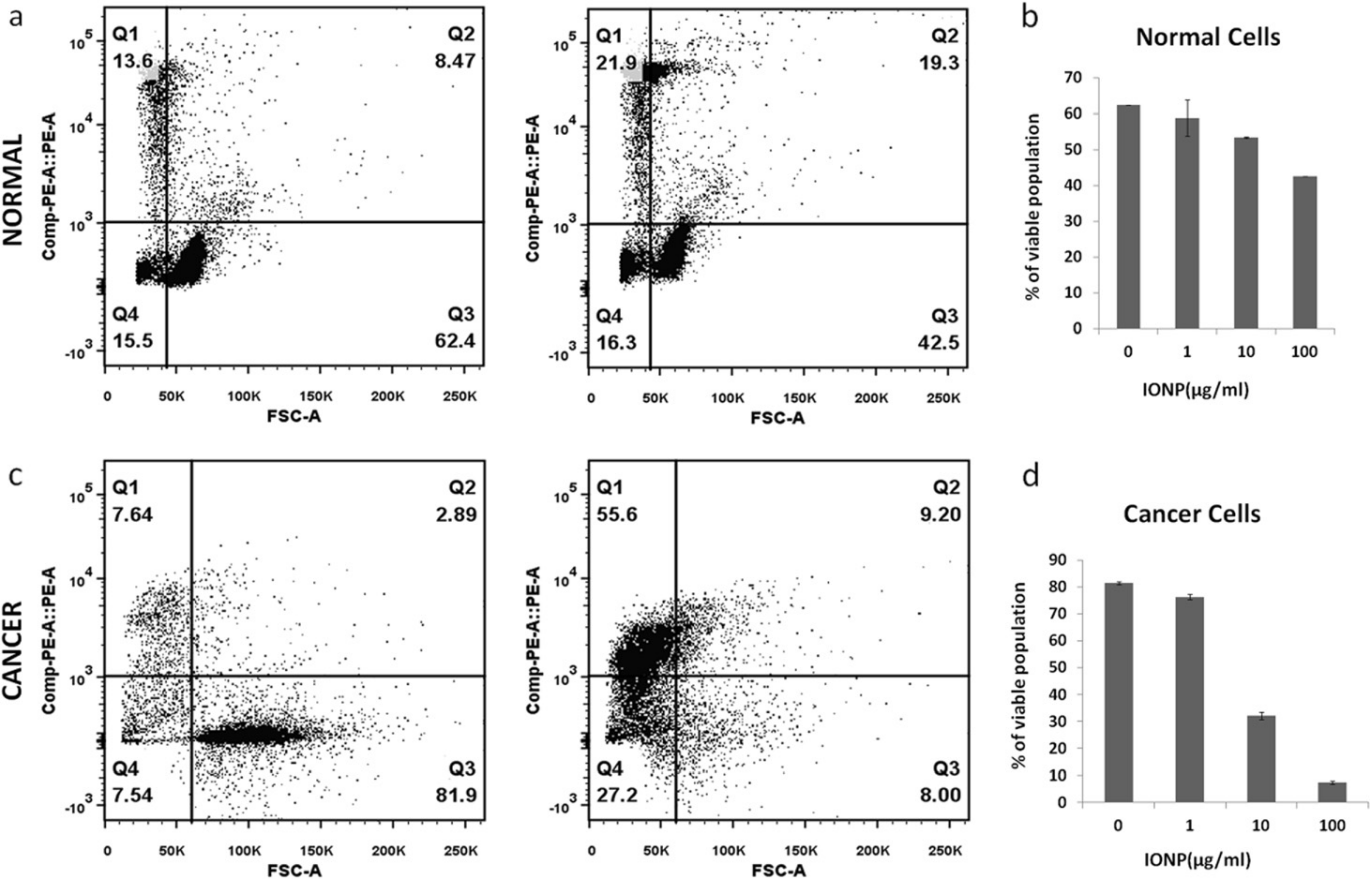
**Comparison of cellular viability in IONP treated cells using Propidium Iodide (PI) uptake.** Panels **a** and **c** show the dot plot of Forward scatter (FSC)- CompPE-A::PE of normal cells (top panel) and cancer cells (bottom panel) in absence (left) and presence (right) of 100 μg/ml IONP treatment. In the dot plot, population are divided into four quadrants (Q1-Q4). Q3 is the PI negative or viable population and whereas Q2 and Q1 are PI positive or dead population of cells. **b** and **d** show effect of different dose of IONP on viable or Q3 population in bar-graph. Viability of normal cells is not much affected by IONP treatment but viability significantly decreases in cancer cells.

**Figure 5:**
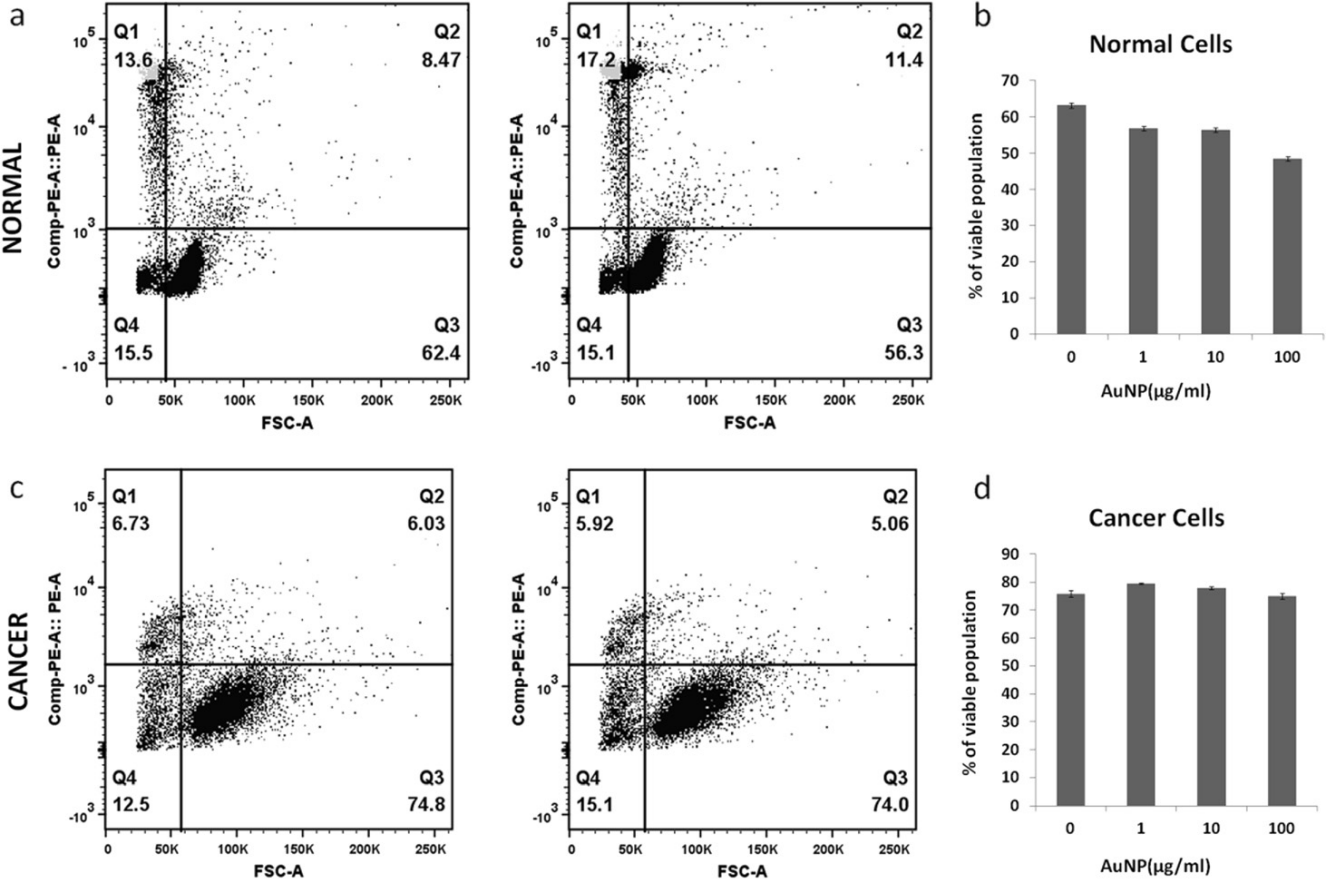
**Comparison of cellular viability in AuNP treated cells using Propidium Iodide (PI) uptake.** Panels **a** and **c** shows the dot plot of Forward scatter (FSC)- CompPE-A::PE of normal cells (top panel) and cancer cells (bottom panel) in absence (left) and presence (right) of 100 μg/ml of AuNP treatment. In the dot plot, population are divided into four quadrants (Q1-Q4). Q3 is the PI negative or viable population and whereas Q2 and Q1 are PI positive or dead population of cells. Panels **b** and **d** showed effect of different dose of AuNP on viable or Q3 population in bar-graph representation. Viability of neither normal cells nor cancer cells was much affected by AuNP treatment.

Propidium Iodide (PI) was used to study cellular viability. Figure 4 included the dot plot of Forward scatter-PI showing cells after treatment with different doses of nanoparticles. The population was divided into four quadrants (Q1-Q4), Q3 represents population of the viable cells whereas Q1and Q2 was non-viable cells. In Figure 4(a and c), PI uptake was plotted against FSC (forward scattering) to monitor the IONP response. When compared with normal cells (Figure 4a) cancer cells (Figure 4c) show much higher degree of response (see quadrant Q3 for normal and cancer cells for IONP at high dose). Figure 4b & d shows graphical comparison of the IONP dose response for normal and cancer cells. While for normal cells there was a subdued response with no clear cut dose dependence, a gradual loss of viability was observed for cancer cells as the IONP concentration was increased gradually from 1 μg/ml to 100 μg/ml. Figure 5 respectively represents data similar to Figure 4, excepting that in this case AuNP is shown instead of IONP. It follows that AuNP did not cause any significant loss in viability for either cancer or normal cells at lower dose. At higher dose there was however some effect, though not as conspicuous as IONP.

### ΔΨ changes in response to Nanoparticles/SMF

#### Depolarisation of cancer cells in response to nanoparticles

**Figure 6:**
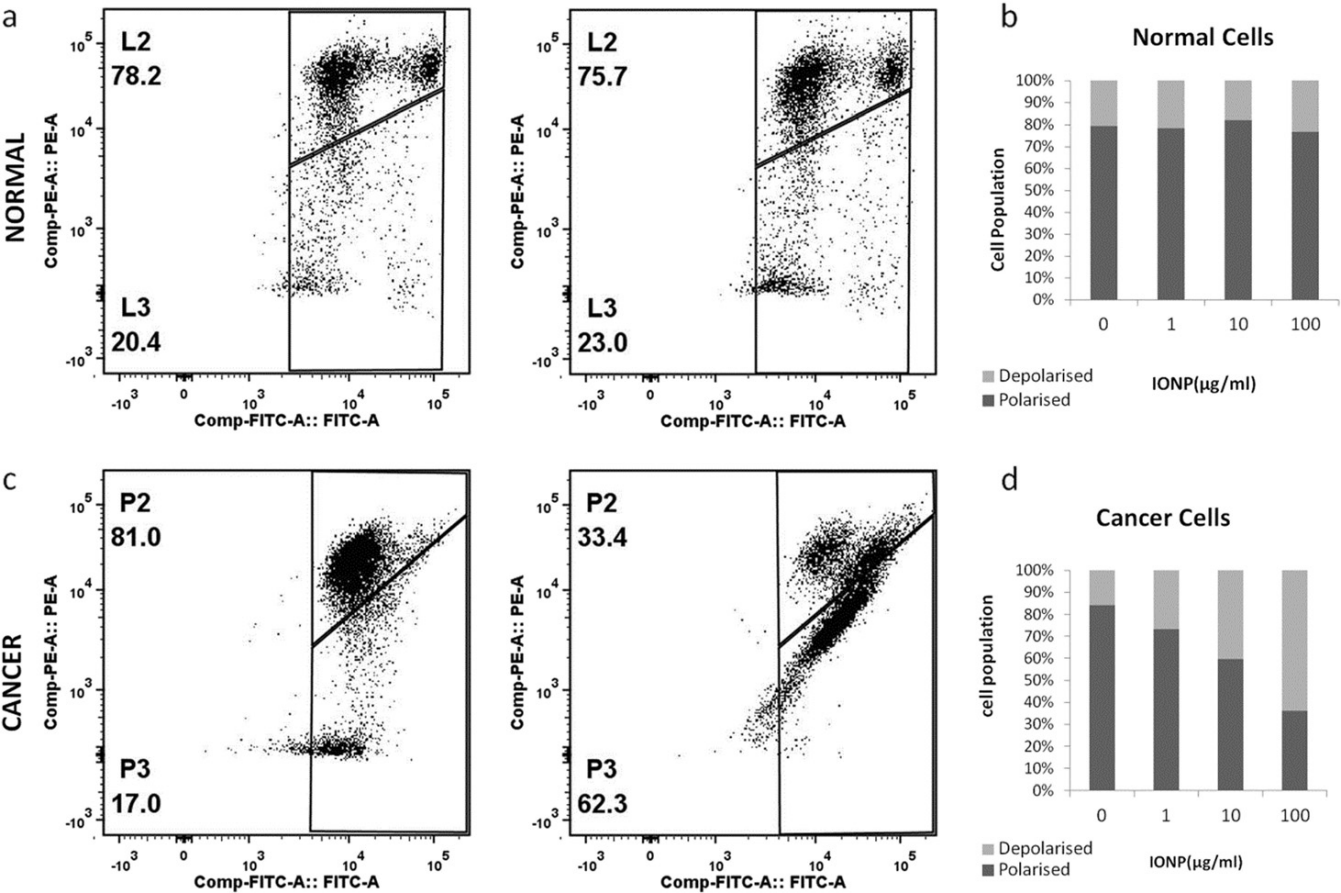
**Sensitivity of ΔΨ of IONP treatment.** ΔΨ was measured using JC-1 probe. Panels **a** and **c** shows the dot plot of FITC –PE channel of normal cells (top panel) and cancer cells (bottom panel) in absence (left panel) and presence (right panel) of 100 μg/ml of IONP. L2 and P2 indicate population with polarised ΔΨ and L3 and P3 indicate depolarised population in case of normal cells and cancer cells respectively. Panels **b** and **d** show the effect of different dose of IONP as bar-graph. P3 or depolarised population is gradually increases with dose of IONP.

**Figure 7:**
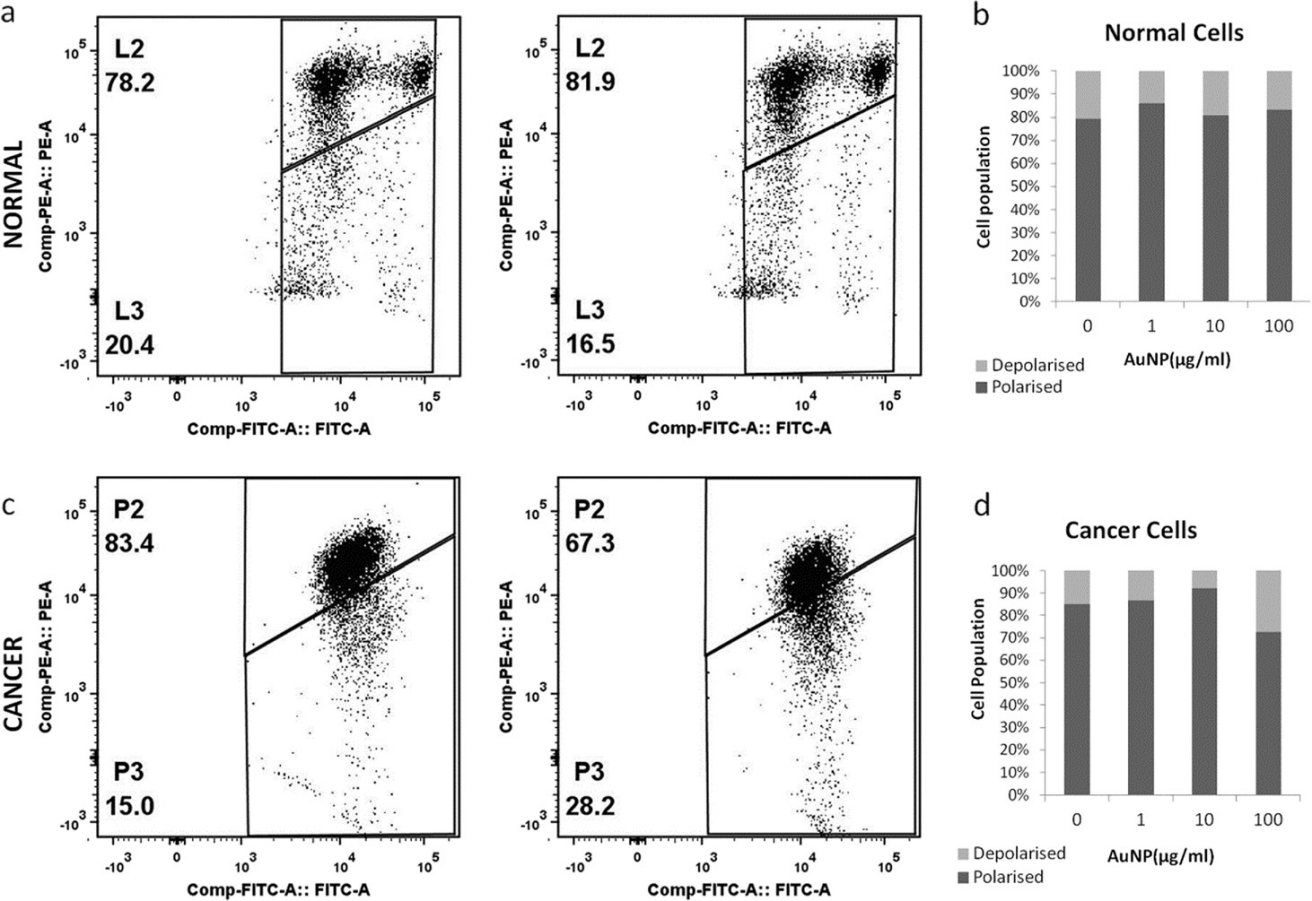
**Insensitivity of ΔΨ of AuNP treatment.** ΔΨ was measured using JC-1 probe. Panels **a** and **c** shows the dot plot of FITC –PE channel of normal cells (top panel) and cancer cells (bottom panel) in absence (left panel) and presence (right panel) of 100 μg/ml of AuNP. L2 and P2 indicate population with polarised ΔΨ and L3 and P3 indicate depolarised population in case of normal cells and cancer cells respectively. Panels **b** and **d** show the effect of different dose of AuNP as bar-graph. AuNP did not affect ΔΨ of either normal cells or cancer cells conspicuously.

The effect of NP on membrane potential for cancer cells and normal cells was compared using JC-1 probe. In polarised cells JC-1 aggregates were formed which gave fluorescence at both FITC and PE channel whereas in depolarised cells JC-1 monomers were present which gave only FITC fluorescence. Both cell types were treated with three different dose of IONP as shown in Figure 6. Polarised population was gated as L2 and P2 and depolarised population as L3 and P3 in case of normal cells and cancer cells respectively. The results of the JC-1 experiment were also shown graphically besides. Similarly effect of AuNP on both normal and cancer cells was shown in Figure 7. Neither AuNP, nor IONP do not altered ΔΨ of normal cells, but IONP caused significant depolarisation of cancer cells (reflecting lowering of the dimer monomer ratio) (see Figure 6). AuNP did not significantly affect ΔΨ of either cancer or normal cells (see Figure 7). There was also a dose-dependent response found in IONP treated cancer cells. With decrease of dose of IONP, depolarisation of cells also decreased (Figure 6d).

#### Differential response of normal and cancer cells

**Figure 8:**
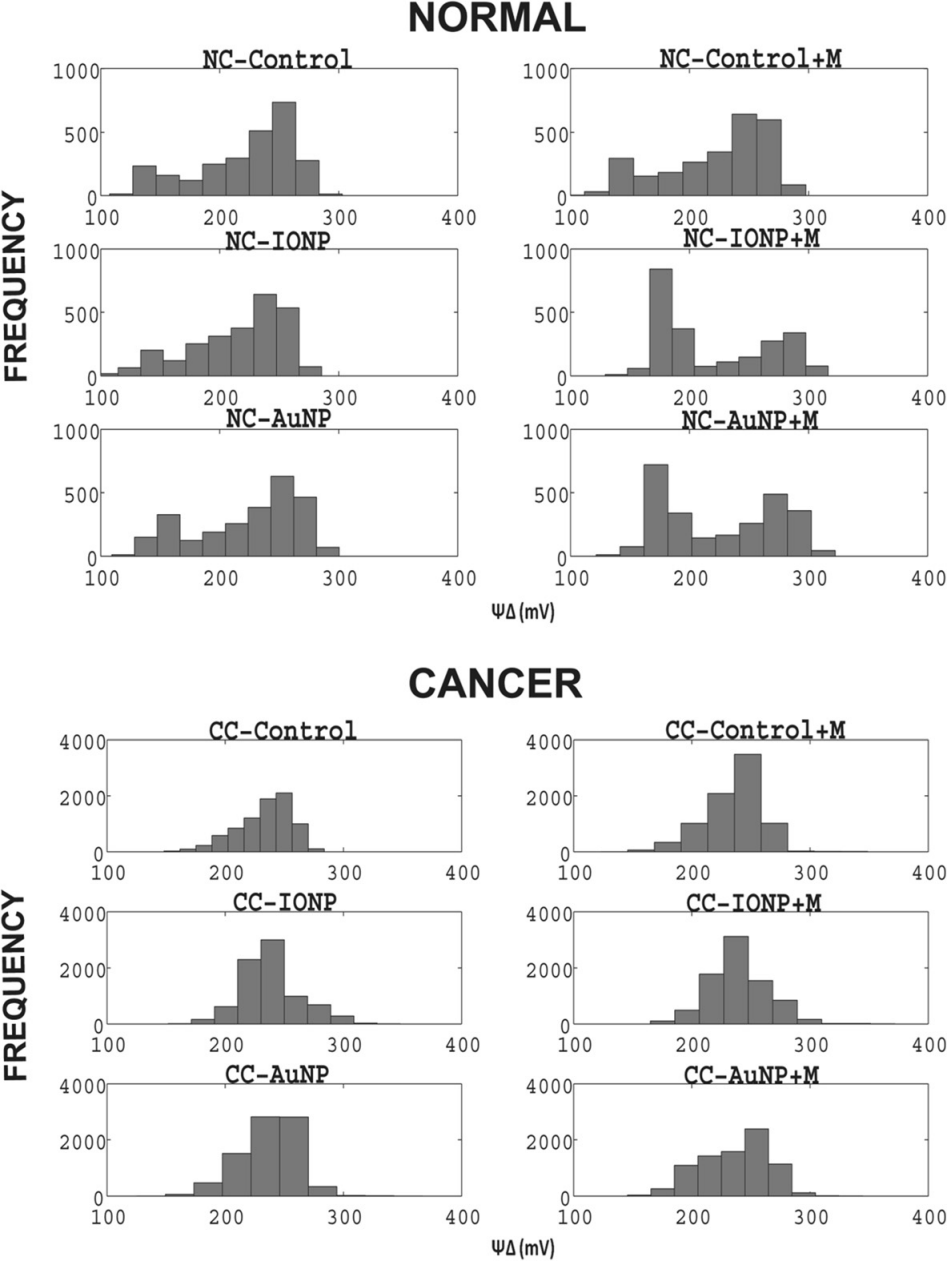
**Alteration of ΔΨ distribution in response to SMF and nanoparticle perturbation.** Top panel compares the changes in ΔΨ distribution of normal cells in presence and absence of SMF. The title of each histogram is given in the figure. Similar panel is shown for cancer cells.

Figure 8 shows the SMF effect in normal and cancer cells with and without nanoparticle. The first striking feature one observes is that in case of normal cells there is a much wider distribution of membrane potential ranging from 150–270 mV, the corresponding range in cancer cells being 200–250 mV. The maximal response was observed in case of normal cells in which the peak maxima shifts leftward that is more depolarised state. This is found in normal cells treated with NP and SMF. Such shift was however minimal in case of cancer cells.

## Discussion

The paper shows that static magnetic field alone, and in combination with nanoparticles can induce changes in DNA integrity and membrane potential. Importantly extent of such changes widely differs in cell types (here normal and cancer cells). In the normal cell type (PBMCs), damage of DNA is minimal and opposite is the case with cancer cells. Diamagnetic properties of biological cells may be responsible for such effects. As shown by [17] that in presence of high intensity magnetic field such as 14 T assemblies and shapes of cell colonies of smooth muscle cells are oriented in the direction of field. However, effect on cellular functions was not studied by the same group. In the present work, we report the effect of low intensity SMF on functionality of biological cell system. Cancer cell is found to lose DNA integrity in the mere presence of SMF. The observation remains true in presence of combination of magnetic field and nanoparticles. The combination effect in turn differs from exclusive effects of nanoparticles, which again is conspicuously more predominant in cancer cells. As diamagnetic effects are expected only at very large field, the other alternative namely quantum coherence in amplifying the relatively low strength field effect cannot be ignored. Such effects are for example reported in some photosynthetic phenomena [27,28].

Table 1, demonstrates the effect of NP,SMF and the effect of NP + SMF on ΔΨ. Only the normal cells shows a limiting depolarization (potential changing from 240 to 180 mV) when challenged by NP + SMF an effect absent in cancer cells.

**Table 1:** **Differential nanoparticle and SMF response of normal and cancer cells**

**Cells**	**Conditions**	**-SMF**		**+SMF**	
		ΔΨ **Range (mV)**	ΔΨ **Maximum (mV)**	ΔΨ **Range (mV)**	ΔΨ **Maximum (mV)**
Normal	Control	140	260	140	240
	IONP	140	240	140	180
	AuNP	140	260	140	180
Cancer	Control	80	240	60	260
	IONP	100	240	100	240
	AuNP	80	250	80	260

The range of ΔΨ values and the ΔΨ at which cells are most populated are shown. The range value is higher for normal cells and the normal cells show a trend to depolarize when SMF and nanoparticles are present.

Unlike the ΔΨ effect, the DNA damage behaviour pattern was slightly different. In this case only the cancer cells showed DNA damage effect when challenged by NP, and NP + SMF (see Figure 1), but not the normal cells. This differential response of cancer and normal cells when challenged by NP and SMF is summarized in Table 2.

**Table 2:** **Nanoparticle and SMF as cell discriminators**

		**NP**	**SMF + NP**
ΔΨ	Normal	**-**	**+**
	Cancer	+	-
**DNA Damage**	Normal	-	-
	Cancer	++	+

The table summarizes cancer cell and normal cell discrimination by two agents namely SMF and NP. The discrimination patterns are based on two cellular targets ΔΨ and DNA damage.

The mechanism of the effect observed in Table 2, needs additional insights which presently can be put forward at postulation level. Collapse of membrane potential, by classical chemisomotic argument would be equivalent to generation of dissipated heat. A local heat shock response in turn [29] would provide a protection to DNA damage. This might be the route taken by normal cells as we find limiting depolarization but absence of any significant DNA damage in such cells. The cancer cells on the other hand somehow resist depolarization by SMF (see Table 1). This is despite the well-known fact that cancer cells have a higher propensity to uptake NP. Interestingly, the Table 1 result, look like a normal cell and cancer cell discriminator. Whether similar results hold good for adhering cells also needs to be probed. Similar conclusions is approved by Figure S2a and Figure S2b in the Additional file 1.

The other result that may draw attention is the IONP induced genotoxicity in cancer cells that is partially reversed by static magnetic field. Whether super- paramagnetic nature of NP that is taken up by cells, causes this difference remains an open question. The result may add an element of manoeuvrability of IONP’s that is already accepted as a theranostic agent [30].

Lastly, the NP induced damages and their modulation by SMF may be helpful for theranostic use of magnetic nanoparticles. AuNP, at high dose also assumes magnetic moment [22] and this is in accordance with some of the positive high dose effect seen with AuNP.

The present model system is a prescription for exploring toolboxes discriminating normal and cancer cells. It can also provide on how the effect of nanoparticle can be modulated by static magnetic field, even when nanoparticle is of non-magnetic in nature. The other point we would like to note here is a negative report by a decade old report by World Health Organization [31]. The report indicated that static and low frequency magnetic field has no significant effect on health hazard. One may wonder whether this result contradicts the result in this paper. We showed that normal cells are relatively unaffected and this indication is consistent with the present the report. What is additionally expected is that the field is toxic for cancer cells, which indirectly implies no hazard (and in fact positive effects) on health. This safety issue may be important in the general context of nanomedicine as wider use of magnetic manipulation of nanoscale objects is expected for drug delivery and theranostics in future.

## Conclusion

- SMF enhances DNA damage of cancer cells, but not for normal cells
- Iron oxide nanoparticles caused DNA damage and loss of cellular viability, such effects seen with AuNP only at higher concentration.
- SMF sensitivity of membrane potential was higher for normal cells, when they were challenged by NPs and reverse otherwise.
- A simple potential collapse mediated heat generation, and the consequent induction of heat shock response may explain this marginally higher DNA protection machinery for normal cells. This hypothesis is partially validated by two facts (i) Cancer cells show higher DNA damage (ii) they show lesser depolarization when they were challenged by a combination of nanoparticles and SMF.
- A simple potential collapse mediated heat generation, and the consequent induction of heat shock response may explain this marginally higher DNA protection machinery for normal cells. This hypothesis is partially validated by two facts (i) Cancer cells show higher DNA damage (ii) they show lesser depolarization when they were challenged by a combination of nanoparticles and SMF.

## Additional file

Additional file 1: Figure S1. Characterisation of nanoparticles: Panel a, Hydrodynamic size distribution of AuNP; b, SEM images of AuNP shows well monodisperse particles of size 30 nm. c, SPR of AuNP shows absorption maxima at 520nm and zeta potential distribution of gold nano-colloids shows mean zeta potential is -41.2 mV. d, Hydrodynamic size distribution of IONP. e, SEM images of IONP shows particles size of 70nm approximately and f, shows the zeta potential distribution of IONP with mean surface charge is -48mV. **Figure S2a.** SMF effect on ΔΨ of cancer cells. The details of image analysis are given in Methods section. Top panels show the effect of IONP (high dose) on cells and Bottom panels shows the effect of AuNP on cells in presence (green color) and absence (red color) of SMF. In the top panels, images show the SMF has no effect on morphology and ΔΨ of IONP treated cancer cells. But the bottom panels of images show that SMF do have some effect on ΔΨ of AuNP treated cells as cells shows leftward shift. **Figure S2b.** SMF effect on ΔΨ of normal cells. The details of image analysis are given in Methods section. Top panels show the effect of IONP (high dose) on cells and bottom panels show the effect of AuNP on cells in presence (green color) and absence (red color) of SMF. Both the panel of images show that SMF has affected the ΔΨ of treated cells but not the morphology. In both cases, ΔΨ shifts towards depolarised state.

## References

[1] Mesnil M, Yamasaki H, Balmain A, Barrett JC, Moses H (1993). Cell‐cell communication and growth control of normal and cancer cells: Evidence and hypothesis. Mol Carcinog.

[2] Tesniere A, Panaretakis T, Kepp O, Apetoh L, Ghiringhelli F, Zitvogel L, Kroemer G (2007). Molecular characteristics of immunogenic cancer cell death. Cell Death Differ.

[3] Jain RK (1987). Transport of molecules in the tumor interstitium: a review. Cancer Res.

[4] Kievit FM, Zhang M (2011). Cancer nanotheranostics: improving imaging and therapy by targeted delivery across biological barriers. Adv Mater.

[5] Brannon-Peppas L, Blanchette JO (2012) Nanoparticle and targeted systems for cancer therapy. Adv Drug Deliv Rev ᅟ:ᅟ 10.1016/j.addr.2004.02.014 15350294

[6] Cho MH, Lee EJ, Son M, Lee J-H, Yoo D, Kim J-w, Park SW, Shin J-S, Cheon J (2012) A magnetic switch for the control of cell death signalling in in vitro and in vivo systems. Nat Materᅟ:ᅟ 10.1038/nmat3430 23042417

[7] Hathaway HJ, Butler KS, Adolphi NL, Lovato DM, Belfon R, Fegan D, Monson TC, Trujillo JE, Tessier TE, Bryant HC (2011). Detection of breast cancer cells using targeted magnetic nanoparticles and ultra-sensitive magnetic field sensors. Breast Cancer Res.

[8] Jordan A, Scholz R, Maier-Hauff K, Johannsen M, Wust P, Nadobny J, Schirra H, Schmidt H, Deger S, Loening S (2001). Presentation of a new magnetic field therapy system for the treatment of human solid tumors with magnetic fluid hyperthermia. J Magn Magn Mater.

[9] Patra HK, Banerjee S, Chaudhuri U, Lahiri P, Dasgupta AK (2007). Cell selective response to gold nanoparticles. Nanomedicine.

[10] Gkanas EI (2013). In vitro magnetic hyperthermia response of iron oxide MNP’s incorporated in DA3, MCF-7 and HeLa cancer cell lines. Cent Eur J Chem.

[11] Huff TB, Tong L, Zhao Y, Hansen MN, Cheng J-X, Wei A (2007). Hyperthermic effects of gold nanorods on tumor cells. Nanomedicine.

[12] Miyakoshi J (2006). The review of cellular effects of a static magnetic field. Sci Technol Adv Mater.

[13] Bhabra G, Sood A, Fisher B, Cartwright L, Saunders M, Evans WH, Surprenant A, Lopez-Castejon G, Mann S, Davis SA (2009). Nanoparticles can cause DNA damage across a cellular barrier. Nat Nanotechnol.

[14] Brigger I, Dubernet C, Couvreur P (2012) Nanoparticles in cancer therapy and diagnosis. Adv Drug Deliv Rev ᅟ:ᅟ 10.1016/s0169-409x(02)00044-3 12204596

[15] Syng-ai C, Basu Baul TS, Chatterjee A (2002). Antiproliferative and cytotoxic effect of a novel organotin compound on mammalian cells both in vitro and in vivo. Mutat Res/Genet Toxicol Environ Mutagen.

[16] Koren HS, Anderson SJ, Larrick JW (1979). In vitro activation of a human macrophage-like cell line. Nature.

[17] Iwasaka M, Miyakoshi J, Ueno S (2003). Magnetic field effects on assembly pattern of smooth muscle cells. In Vitro Cell Dev Biol Anim.

[18] DASGUPTA AK, Raja SO (2013). Static Magnetic Field Induced Differential Fluorescence Emission.

[19] Frens G (1973). Controlled nucleation for the regulation of the particle size in monodisperse gold suspensions. Nature.

[20] Kimling J, Maier M, Okenve B, Kotaidis V, Ballot H, Plech A (2006). Turkevich method for gold nanoparticle synthesis revisited. J Phys Chem B.

[21] Turkevich J, Stevenson PC, Hillier J (1951). A study of the nucleation and growth processes in the synthesis of colloidal gold. Discuss Faraday Soc.

[22] Banerjee S, Raja S, Sardar M, Gayathri N, Ghosh B, Dasgupta A (2011). Iron oxide nanoparticles coated with gold: enhanced magnetic moment due to interfacial effects. J Appl Phys.

[23] Fuss IJ, Kanof ME, Smith PD, Zola H (2009). Isolation of whole mononuclear cells from peripheral blood and cord blood. Curr Protoc Immunol.

[24] Singh NP, McCoy MT, Tice RR, Schneider EL (1988). A simple technique for quantitation of low levels of DNA damage in individual cells. Exp Cell Res.

[25] Reers M, Smith TW, Chen LB (1991). J-aggregate formation of a carbocyanine as a quantitative fluorescent indicator of membrane potential. Biochemistry.

[26] Yeh C-JG, Hsi B-L, Page Faulk W (1981). Propidium iodide as a nuclear marker in immunofluorescence. II. Use with cellular identification and viability studies. J Immunol Methods.

[27] Heinen U, Poluektov O, Stavitski E, Berthold T, Ohmes E, Schlesselman SL, Golecki JR, Moro GJ, Levanon H, Thurnauer MC (2004). Magnetic-field-induced orientation of photosynthetic reaction centers, as revealed by time-resolved D-band electron paramagnetic resonance of spin-correlated radical pairs. II. Field dependence of the alignment. J Phys Chem B.

[28] Hoff A (1981). Magnetic field effects on photosynthetic reactions. Q Rev Biophys.

[29] Chiu H-Y, Tsao L-Y, Yang R-C (2009). Heat-shock response protects peripheral blood mononuclear cells (PBMCs) from hydrogen peroxide-induced mitochondrial disturbance. Cell Stress Chaperones.

[30] Shubayev VI, Pisanic TR, Jin S (2009). Magnetic nanoparticles for theragnostics. Adv Drug Deliv Rev.

[31] Repacholi MH, Greenebaum B (1999). Interaction of static and extremely low frequency electric and magnetic fields with living systems: health effects and research needs. Bioelectromagnetics.

