# Supplementary material for: Modulation of cytotoxic and genotoxic effects of nanoparticles in cancer cells by external magnetic field
Source: Cancer Nanotechnol. 2014 Jun 26;5(1):2. doi: 10.1186/s12645-014-0002-x (PMC4631716; doi:10.1186/s12645-014-0002-x)
Supplement: Additional file 1: Figure S1. — Characterisation of nanoparticles: Panel a, Hydrodynamic size distribution of AuNP; b, SEM images of AuNP shows well monodisperse particles of size 30 nm. c, SPR of AuNP shows absorption maxima at 520nm and zeta potential distribution of gold nano-colloids shows mean zeta potential is -41.2 mV. d, Hydrodynamic size distribution of IONP. e, SEM images of IONP shows particles size of 70nm approximately and f, shows the zeta potential distribution of IONP with mean surface charge is -48mV. Figure S2a. SMF effect on ΔΨ of cancer cells. The details of image analysis are given in Methods section. Top panels show the effect of IONP (high dose) on cells and Bottom panels shows the effect of AuNP on cells in presence (green color) and absence (red color) of SMF. In the top panels, images show the SMF has no effect on morphology and ΔΨ of IONP treated cancer cells. But the bottom panels of images show that SMF do have some effect on ΔΨ of AuNP treated cells as cells shows leftward shift. Figure S2b. SMF effect on ΔΨ of normal cells. The details of image analysis are given in Methods section. Top panels show the effect of IONP (high dose) on cells and bottom panels show the effect of AuNP on cells in presence (green color) and absence (red color) of SMF. Both the panel of images show that SMF has affected the ΔΨ of treated cells but not the morphology. In both cases, ΔΨ shifts towards depolarised state. [file 12645_2014_2_MOESM1_ESM.docx]

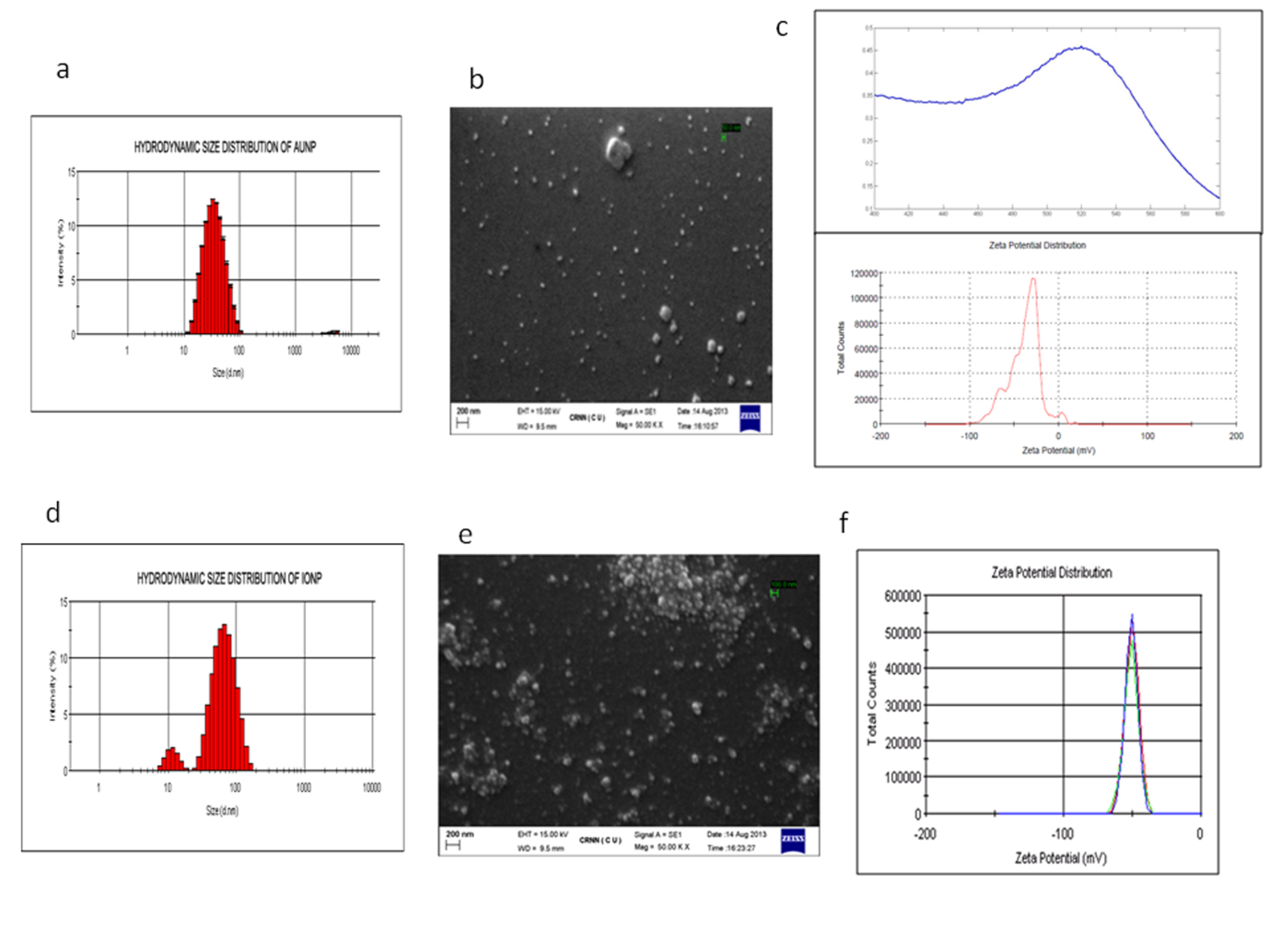


Figure S1: Characterisation of nanoparticles: Panel a, Hydrodynamic size distribution of AuNP; b, SEM images of AuNP shows well monodisperse particles of size 30nm. c, SPR of AuNP shows absorption maxima at 520nm and zeta potential distribution of gold nano-colloids shows mean zeta potential is -41.2mV. d, Hydrodynamic size distribution of IONP. e, SEM images of IONP shows particles size of 70nm approximately and f, shows the zeta potential distribution of IONP with mean surface charge is -48mV


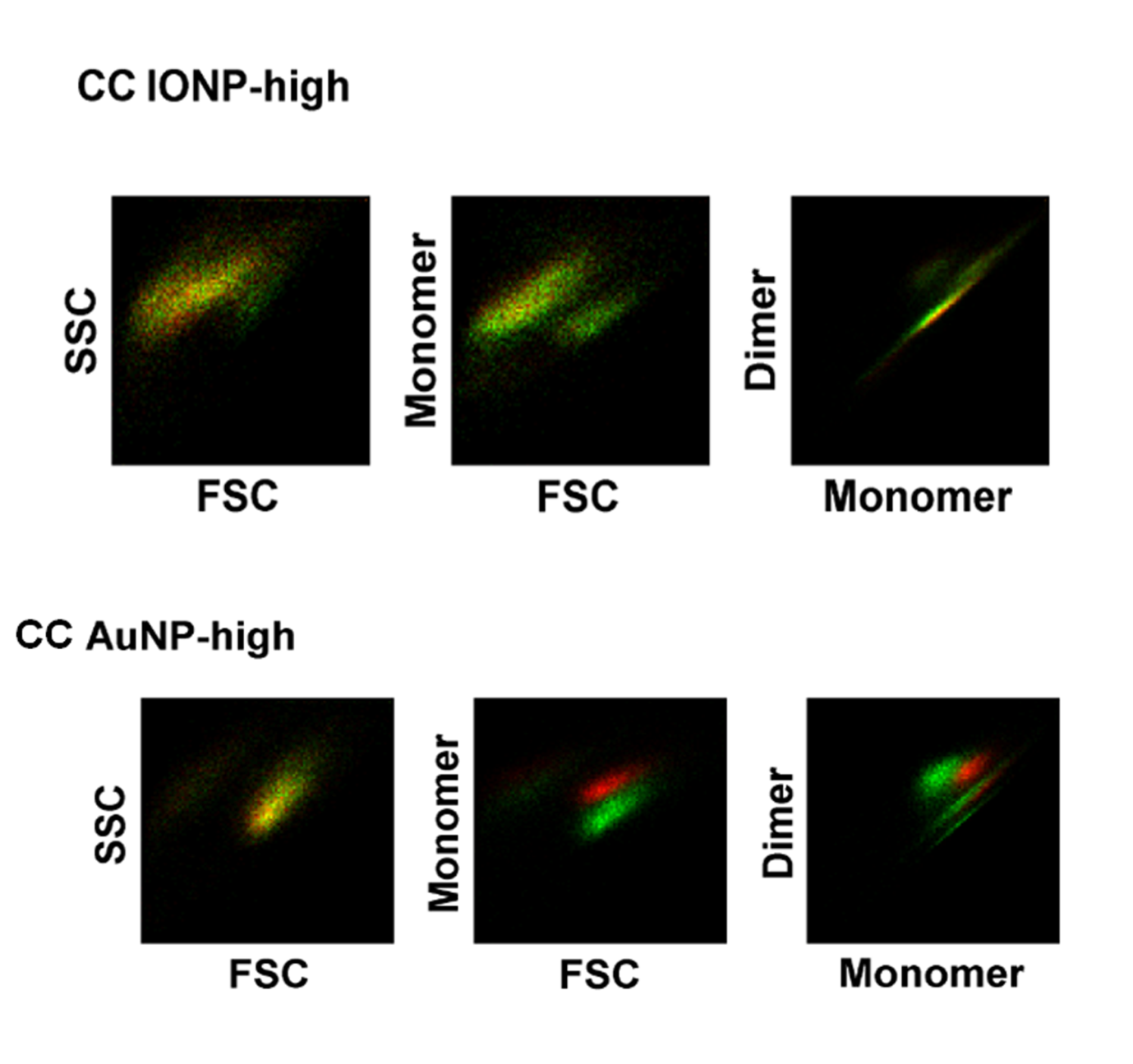


Figure S2a: SMF effect on ΔΨ of cancer cells. The details of image analysis are given in materials and method section. Top panels show the effect of IONP (high dose) on cells and Bottom panels shows the effect of AuNP on cells in presence (green color) and absence (red color) of SMF. In the top panels, images show the SMF has no effect on morphology and ΔΨ of IONP treated cancer cells. But the bottom panels of images show that SMF do have some effect on ΔΨ of AuNP treated cells as cells shows leftward shift.


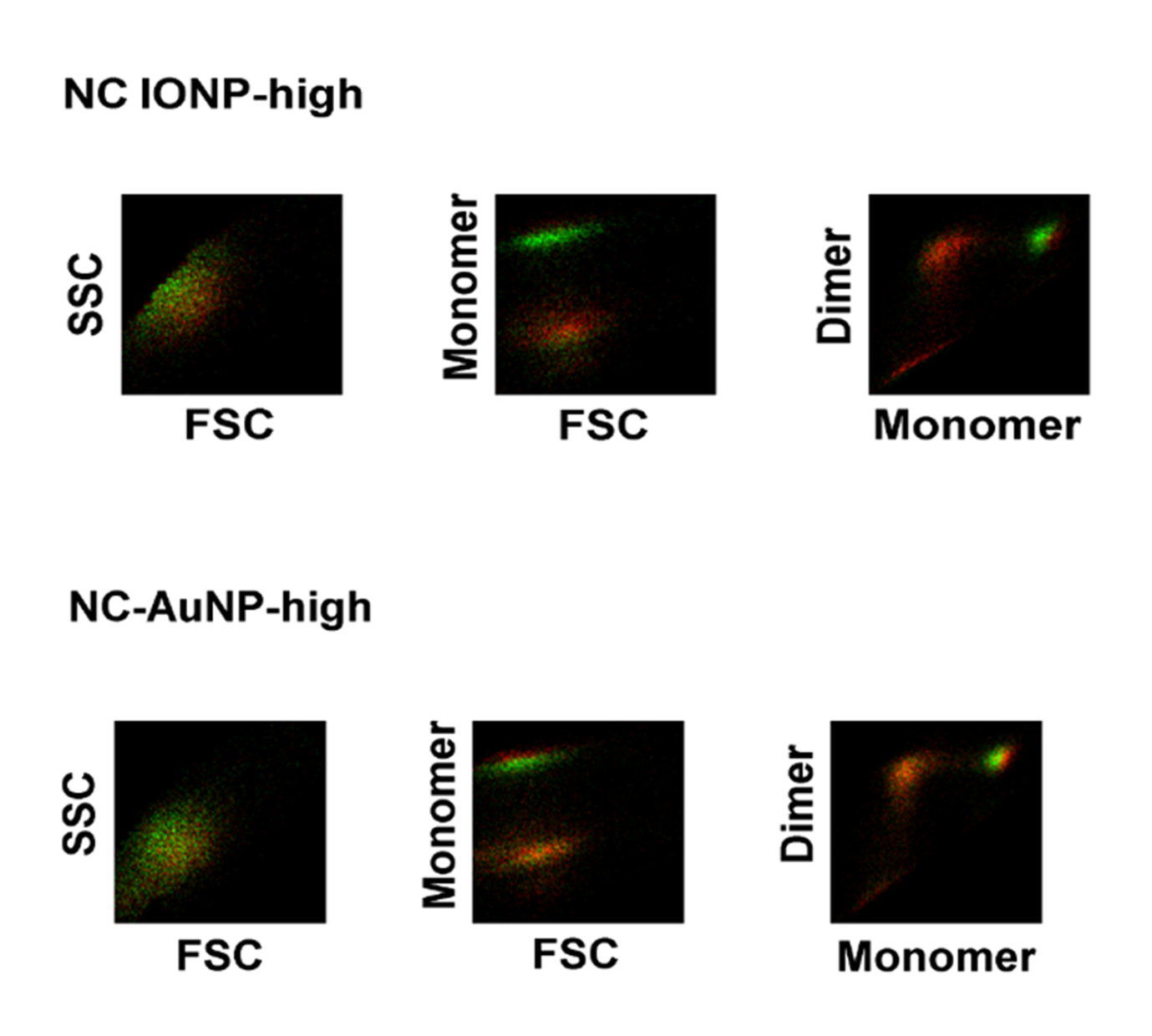


Figure S2b: SMF effect on ΔΨ of normal cells. The details of image analysis are given in materials and method section. Top panels show the effect of IONP (high dose) on cells and bottom panels show the effect of AuNP on cells in presence (green color) and absence (red color) of SMF. Both the panel of images show that SMF has affected the ΔΨ of treated cells but not the morphology. In both cases, ΔΨ shifts towards depolarised state.
